# Human immunodeficiency virus-positive patients with parotid gland disease referred for otolaryngology consultation: a case series

**DOI:** 10.1016/j.bjorl.2023.101339

**Published:** 2023-10-09

**Authors:** Toshiyuki Akama, Takeshi Tsuda, Kazuya Takeda, Hiroshi Nishimura

**Affiliations:** a National Hospital Organization Osaka National Hospital, Department of Otorhinolaryngology, Osaka, Japan; b Osaka University Graduate School of Medicine, Department of Otorhinolaryngology, Head and Neck Surgery, Osaka, Japan

## Introduction

Human Immunodeficiency Virus (HIV) infection causes serious diseases of the ear, nose, and throat.^1^ The occurrence of Benign Lymphoepithelial Cysts (BLEC) in the salivary gland region is considered to be a specific and early manifestation of HIV infection.^2^ Although BLEC in the salivary gland region is well recognized, there are no reports of a comprehensive evaluation of parotid gland diseases associated with HIV. Otorhinolaryngologists play an important role in HIV treatment; however, very few otorhinolaryngology clinics routinely treat patients with HIV. Thus, the aim of this study was to review the data of patients with HIV who were referred from the Department of Infectious Diseases to the Department of Otolaryngology at our hospital for treatment of symptoms related to the parotid gland.

## Case report

We retrospectively analyzed the data of patients referred between 2016 and 2021. The included patients comprised 13 males and 3 females (median age: 47 [36–74] years). Four patients had bilateral parotid swelling and 12 had unilateral parotid swelling. All patients underwent ultrasonography; however, only 10 underwent fine-needle aspiration. Three patients underwent Computed Tomography (CT) and six underwent Magnetic Resonance Imaging (MRI). Three patients had an inflammatory disease, four had benign tumors, and five had other diseases (Table 1).

**Table 1:** Clinical demographics.

Nº	Diagnosis	Age	Sex	Medication	CD4 (/μL)	CD8 (/μL)	HR (/mL)
1	WT	40	M	Lamivudine, Raltegravir	744	750	<20
2	PA	46	F	Elvitegravir, Emtricitabine, Tenofovir, Cobicistat	934	662	<20
3	WT	59	M	Emtricitabine, Tenofovir, Rarltegravir	304	490	<20
4	PA	38	M	Bictegravir, Emtricitabine, Tenofovir	47	457	46
5	PC	59	M	Doravirine, Darunavir, Ritonavir	307	458	<20
6	PC	55	M	Dolutegravir, Emtricitabine, Tenofovir	468	358	<20
7	Normal	36	M	Elvitegravir, Emtricitabine, Tenofovir, Cobicistat	358	442	<20
8	Normal	40	M	Dolutegravir, Abacavir, Lamivudine	379	565	<20
9	Normal	51	M	Dolutegravir, Abacavir, Lamivudine	821	536	<20
10	Normal	64	M	Darunavir, Ritonavir, Abacavir, Lamivudine	758	458	<20
11	BLEC	38	M	Darunavir, Cobicistat, Emtricitabine, Tenofovir	153	1038	98,900
12	BLEC	74	M	Darunavir, Ritonavir, Abacavir, Lamivudine	430	980	81,400
13	Pneumoparotid	50	M	Darunavir, Ritonavir, Abacavir, Lamivudine	612	724	<20
14	AP	44	M	Darunavir, Cobicistat, Emtricitabine, Tenofovir	253	672	44
15	AP	45	F	Raltegravir, Darunavir, Ritonavir, Etravirine	534	1003	<20
16	AP	48	F	Raltegravir, Darunavir, Ritonavir, Etravirine	530	1078	<20

Four patients had bilateral parotid swelling and 12 had unilateral parotid swelling. The diagnoses included four cases of acute parotitis, two of Warthin’s tumor, two of pleomorphic adenoma, two of parotid cyst, one of pneumoparotid, two of benign lymphoepithelial cysts, and four with no abnormal findings.

WT, Warthin’s Tumor; PA, Pleomorphic Adenoma; PC, Parotid Cyst; BLEC, Benign Lymphoepithelial Cyst; AP, Acute Parotitis.

The median white blood cell count of the patients was 9100 (5700–10,700)/μL, the neutrophil count was 6297.2 (2599–7126)/μL, the CD4 (+) cell count was 530 (253–530)/μL, the CD8 (+) cell count was 1003 (672–1078)/μL, and the C-reactive protein level was 0.11 (0.04–4.23) mg/L.

Three patients were diagnosed with acute parotitis without abscesses. In terms of Antiretroviral Therapy (ART), two patients were treated with raltegravir, darunavir, ritonavir, and etravirine, and one with darunavir/cobicistat. Two patients had HIV RNA levels of < 20 copies/mL, whereas the other patient had an HIV RNA level of 46 copies/mL. Although none of the treated patients underwent CT/MRI, ultrasonography showed roughening of the parotid gland. In all cases, patients showed improvement under oral antibiotic administration.

Two patients had Warthin’s tumors and two had pleomorphic adenomas. One patient with Warthin’s tumor and one with pleomorphic adenoma underwent parotidectomy, with no postoperative recurrence. The other two patients who did not undergo surgery were followed-up.

In addition to other diseases, two cases of a single parotid cyst, one case of pneumoparotid, and two cases of BLEC were observed. One of the two patients with BLEC was treated elsewhere. Patients 1 and 2, with BLEC and pneumoparotid, respectively, are described in detail.

### Patient 1

**Figure 1:**
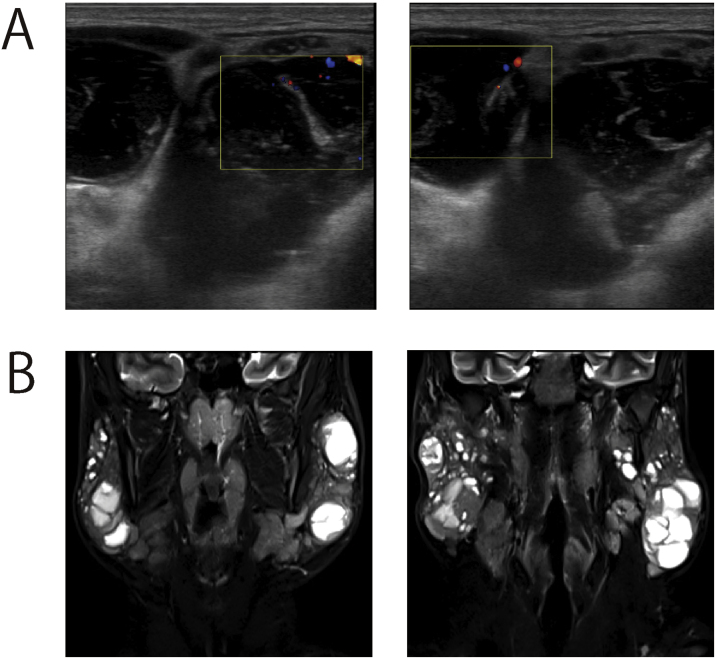
Imaging examination of a patient with benign lymphoepithelial cysts. (A) Ultrasound images. Multiple cystic lesions were detected in both parotid glands. (B) Numerous cystic lesions and no obvious tumors were observed on ultrasound imaging.

Patient 1 was receiving treatment for HIV encephalitis at the Department of Infectious Diseases of our hospital. The patient was referred for examination of an enlarged parotid gland. Ultrasonography revealed multiple cystic lesions in both parotid glands, and cytology revealed lymphocyte infiltration without atypia (Fig. 1A). MRI revealed scattered cystic lesions but no obvious neoplastic lesions. Therefore, the diagnosis of BLEC was made (Fig. 1B). The tumors tended to shrink with ART administration.

### Patient 2

**Figure 2:**
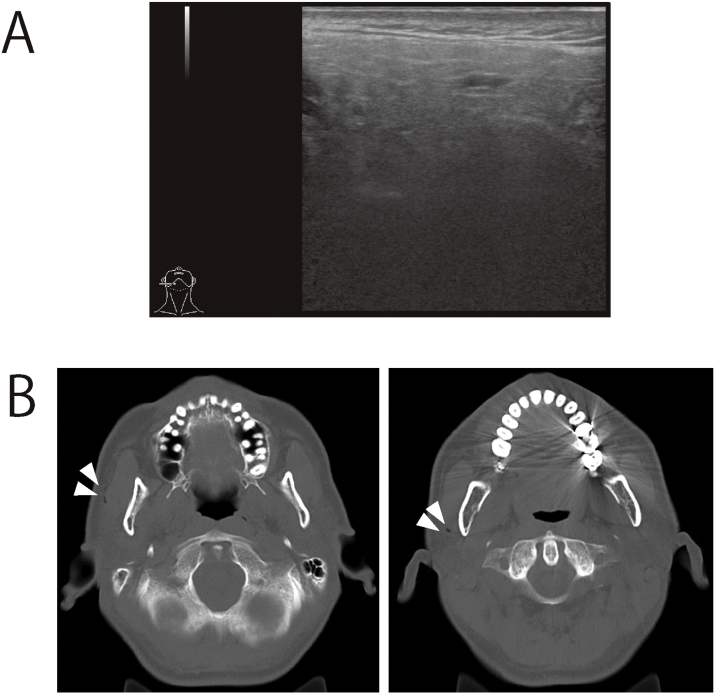
Imaging examination of a patient with pneumoparotid. (A) Ultrasound images. There were no obvious abnormal findings. (B) Gas infiltration was detected in the right parotid gland (white arrows).

Patient 2 presented with a swelling of the right parotid gland. No abnormal findings were observed on neck palpation during the initial examination. Ultrasonography revealed no detectable parotid gland abnormalities. CT was performed to investigate the possibility of parotid salivary stone formation (Fig. 2A). Cervical CT revealed gas infiltration of the gland, which led to a diagnosis of pneumoparotid (Fig. 2B). Both patients are currently being followed-up.

## Discussion

ART reduces the incidence of Acquired Immunodeficiency Syndrome (AIDS) and opportunistic infections in HIV-infected patients, and it helps prevent severe bacterial infections.^3^ In this study, all patients with inflammatory diseases had already been treated with ART; thus, no serious infections were observed. Kaposi's sarcoma and AIDS-related non-Hodgkin's lymphoma are well-known malignancies associated with HIV infection.^4^ However, there have been no reports of benign tumors that are likely to be associated with HIV infection.

BLEC is a well-known parotid disease that is associated with HIV infection. A BLEC is a cyst of the lymphoid tissue that migrates into the parotid gland during fetal life. Although cervical abscesses have been reported in patients with BLEC, the prognosis is generally good. The patient described in this report did not wish to undergo surgery. However, the mass appeared to decrease in size after ART initiation.

Pneumoparotid is a rare disease characterized by the retrograde entry of air into the parotid gland through the parotid duct. Although pneumoparotid often resolves spontaneously, antibiotics are administered to prevent infections in some cases. If recurrence occurs, parotid duct ligation or parotidectomy should be considered.^5^ As the case in the present study was the first occurrence, and the patient recovered spontaneously, no treatment was administered.

## Conclusions

Infections, AIDS-related malignant lymphoma, and BLEC are noteworthy occurrences in HIV-infected patients. However, rare diseases, such as pneumoparotid, also occur. Appropriate examination using CT, MRI, or ultrasonography is important for timely detection and intervention.

## Funding

This study did not receive any specific grants from funding agencies in the public, commercial, or not-for-profit sectors.

## Ethics approval statement

This study was approved by the Ethics Committee of the National Hospital Organization of Osaka National Hospital (nº 21076).

## Patient consent statement

Informed consent was obtained from the patients through an opt-out system via the hospital website.

## Conflicts of interest

The authors declare no conflicts of interest.
